# How to use the Surveillance, Epidemiology, and End Results (SEER) data: research design and methodology

**DOI:** 10.1186/s40779-023-00488-2

**Published:** 2023-10-31

**Authors:** Wen-Qiang Che, Yuan-Jie Li, Chi-Kwan Tsang, Yu-Jiao Wang, Zheng Chen, Xiang-Yu Wang, An-Ding Xu, Jun Lyu

**Affiliations:** 1 Department of Neurosurgery, the First Affiliated Hospital of Jinan University, Guangzhou, 510632 China; 2 Department of Clinical Research, the First Affiliated Hospital of Jinan University, Guangzhou, 510632 China; 3 Planning & Discipline Construction Office, the First Affiliated Hospital of Jinan University, Guangzhou, 510632 China; 4 Clinical Neuroscience Institute, the First Affiliated Hospital of Jinan University, Guangzhou, 510632 China; 5 Department of Pathology, Shanxi Provincial People’s Hospital, Taiyuan, 030012 China; 6 Department of Urology, the First Affiliated Hospital of Jinan University, Guangzhou, 510632 China; 7 Department of Neurology, the First Affiliated Hospital of Jinan University, Guangzhou, 510632 China; 8 Guangdong Provincial Key Laboratory of Traditional Chinese Medicine Informatization, Guangzhou, 510632 China

**Keywords:** Surveillance, Epidemiology, and End results (SEER), Big data, Epidemiology, Methodologies, Study design

## Abstract

In the United States (US), the Surveillance, Epidemiology, and End Results (SEER) program is the only comprehensive source of population-based information that includes stage of cancer at the time of diagnosis and patient survival data. This program aims to provide a database about cancer incidence and survival for studies of surveillance and the development of analytical and methodological tools in the cancer field. Currently, the SEER program covers approximately half of the total cancer patients in the US. A growing number of clinical studies have applied the SEER database in various aspects. However, the intrinsic features of the SEER database, such as the huge data volume and complexity of data types, have hindered its application. In this review, we provided a systematic overview of the commonly used methodologies and study designs for retrospective epidemiological research in order to illustrate the application of the SEER database. Therefore, the goal of this review is to assist researchers in the selection of appropriate methods and study designs for enhancing the robustness and reliability of clinical studies by mining the SEER database.

## Background

The Surveillance, Epidemiology, and End Results (SEER) program is an authoritative source for cancer statistics that President Richard Nixon initiated on January 1, 1973. This program is funded by the National Cancer Institute to provide cancer data to the public for clinical studies with the goal of lowering the cancer burden in the United States (US) [1]. The SEER program collects demographic, clinical, and outcome data on all malignancies diagnosed in representative geographic regions and subpopulations in the US. Originally, there were only 9 initial tumor registries, and now there are 22 US geographic areas participating in the SEER program, encompassing about 48% of the total cancer patients in the US population. Information about the detailed surgical procedures has been included in the program since 1983, and tumor types were also covered from 1998. In addition, specific tumor hallmarks have been included for testicular, breast, and prostate cancers since 2004. Based on the 7th edition of the American Joint Committee on Cancer Staging Manual, SEER data were markedly enriched with tumor grades, invasion/metastasis status (bone, brain, lung, and liver), site-specific variables, and tumor stages. Information about the types of radiotherapy, surgical procedures, and the status of chemotherapy was later included in the program.

**Fig. 1:**
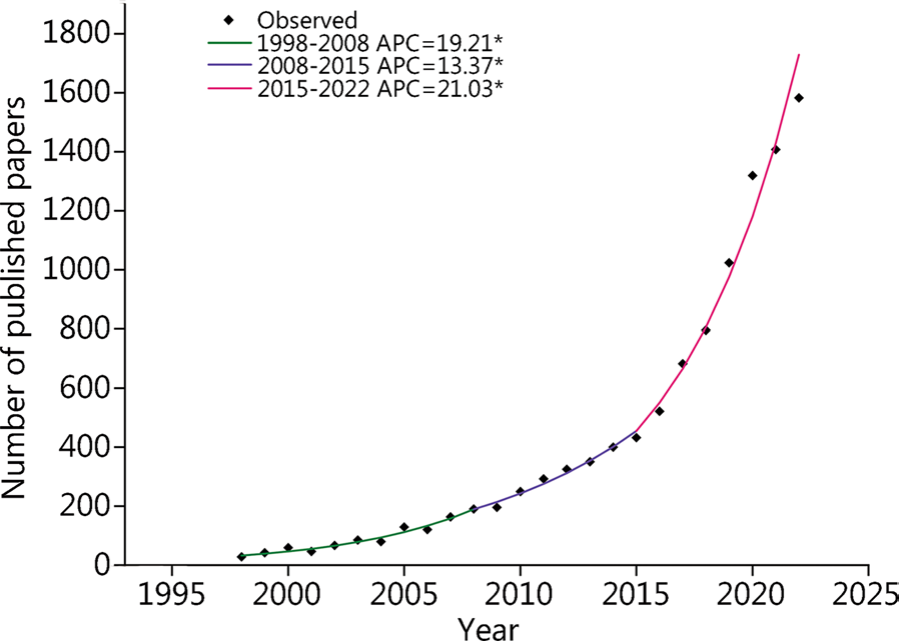
Research articles based on the Surveillance, Epidemiology, and End Results (SEER) (not SEER-Medicare) that had been published in journals from 1998 to 2022 searched by PubMed. The joinpoint analysis program chose the most suitable loglinear regression model to detect calendar years (known as “joinpoints”) with significant changes in APCs, allowing for the minimum number of joinpoints necessary to fit the data. Joinpoint regression analyses detected three segments (1998 – 2008, 2008 – 2015, and 2015 – 2022) that had significant APC changes in the number of published papers. The diamond dots reflect the observed value, whereas the line formed via joinpoint analysis represents the predicted value. The data were assessed on April 23, 2023. Asterisks (*) represent a *P*-value less than 0.05. APC annual percentage change

One of the main targets of the SEER program is to record cancer incidences and mortality rates for the entire US population. To provide insight into the potential etiologies, the program monitors the trends in annual cancer incidence to detect unusual changes in certain cancers that occur in populations stratified by demographic, geographic, and social characteristics. In addition, it facilitates the accumulation of information about disease progression, the identification of prognostic factors, the patterns of healthcare and clinical practices, as well as the variables for determining patient survival quality. As one of the most widely used open-access databases, SEER has facilitated the development of precision medicine and individualized therapies, which could enhance the quality of health care, cut unnecessary costs, improve prevention strategies, and encourage healthy lifestyles at the population level [2–4]. The SEER database can also be used in observational studies and national and local public health programs that could promote health through the prevention and control of diseases [5–7]. Moreover, SEER-based studies have been proven to be useful in the dissection of disease etiologies and have provided guidance for measures that aim to eliminate ethnic disparities [8, 9]. More than 17,000 articles published from 1973 to 2020 used the SEER database as the primary source of data, and more than 86,000 articles referenced SEER in their studies. Figure 1 shows the progressive growth in the number of published articles based on SEER data in PubMed over the past 25 years (1998–2022). Considering that a handy user guide for the application of the SEER database is still lacking, this review aims to discuss the commonly used methodologies and study designs for SEER-based research.

**Fig. 2:**
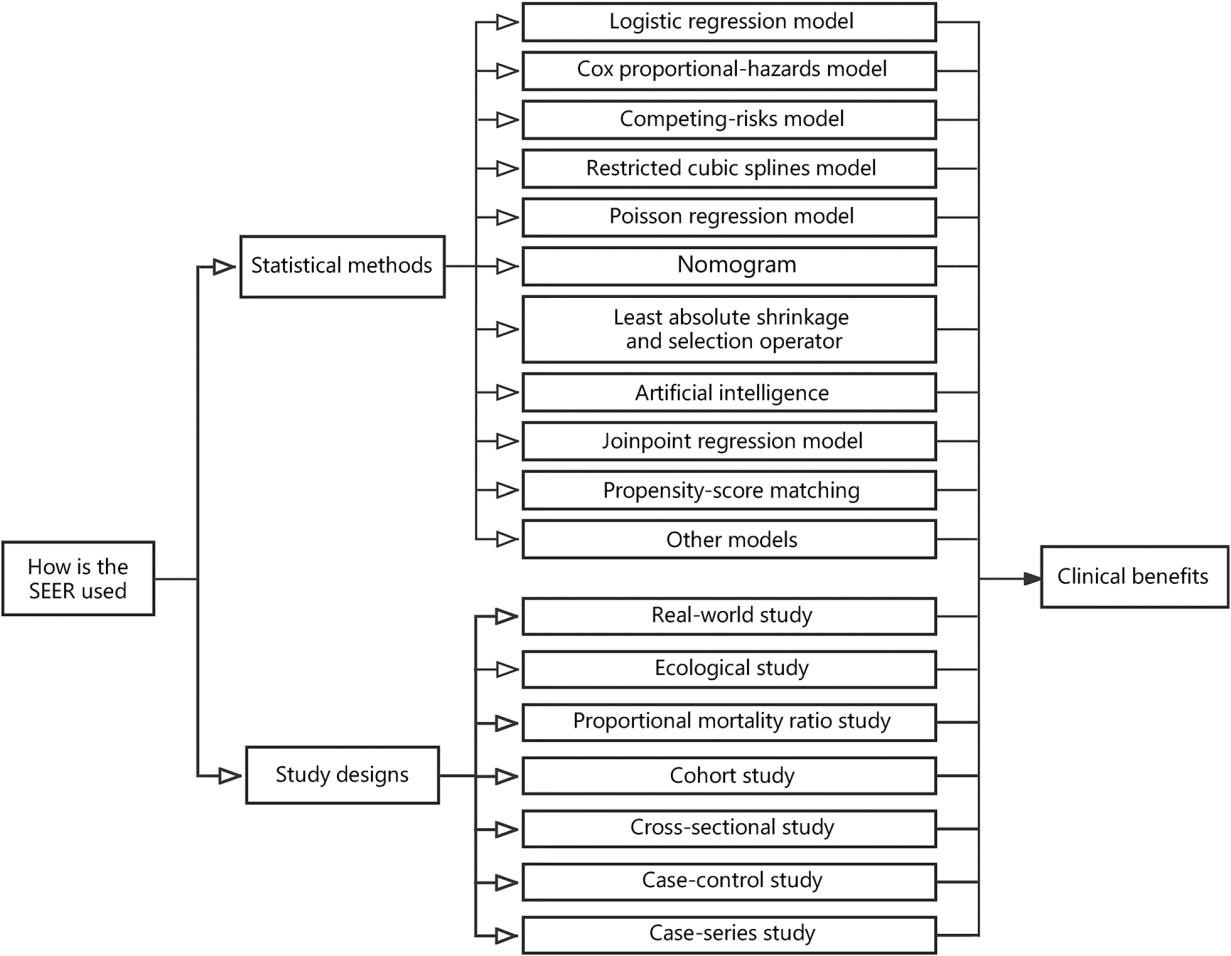
The available methodologies and study designs used in Surveillance, Epidemiology, and End Results (SEER)-based analyses. There are more than 10 analytic methodologies and 7 study designs available for the analysis of the SEER data. The selection of proper study design and analytic methodologies is crucial for utilizing SEER data to generate clinical benefits

Data are of paramount importance in today’s world [10]. In particular, “big data” is thought to have a considerable positive impact on the healthcare system, as in finance and other systems [11]. High degrees of dimensionality, continuous and rapid renewal, scarcity, and irregularity are characteristics of clinical data [12]. To better use big data, it is necessary to overcome various challenges related to technologies, populations, and organizational differences [13]. In addition, identification of the availability of medical databases, data-mining methodologies, and data standardization procedure are essential for successful and reliable clinical and epidemiological studies [14, 15]. For the purpose of facilitating the use of the SEER database, we will discuss the 10 commonly used analytic approaches and 7 study designs. Typical examples will be provided for each topic in order to make it easier for readers to understand the practical application of the SEER database (Fig. 2). SEER updates the database on patient-specific and tumor-specific variables on a regular basis. Therefore, the common variables currently used in the SEER database, including patient demographics, socioeconomic and geographic characteristics, primary tumor locations, tumor morphologies, stages at diagnosis, first-course treatments, follow-up for vital status, causes of death, and other descriptions, are shown in Table 1.

**Table 1:** Commonly used variables in SEER database

Type	Available variable	Cancer type	Description
Sociodemographics	Patient ID	NOS	Personal Identification Number
	Age	NOS	Patient age at diagnosis
	Sex	NOS	Gender in biological terms
	Race/ethnicity	NOS	Physical traits and cultural identity
	Year/Month of diagnosis	NOS	Month of diagnosis is excluded
	Year of birth	NOS	NA
	Marital status	NOS	Marital status at diagnosis
	Insurance	NOS	Excluded since 2022
	Census tract income	NOS	Median household income
Geographic location	Location	NOS	State-county at diagnosis; excluded since 2022
	Registry	NOS	State of registry
	Urban and rural distribution	NOS	Urban and rural areas
Socioeconomics	Socioeconomic status factors	NOS	22 county-level factors reported by American Community Survey County Attributes
Tumor	Site	NOS	ICD-O-3 topography code
	Histology	NOS	ICD-O-3 morphology code
	Sequence	NOS	Sequence of reported tumor
	Biological characteristics	CNS	WHO grade, laterality, size
		Non-CNS	Breast cancer subtype, etc. WHO grade, laterality, size
	Biomarkers	CNS	*IDH*-mutant, *1p*/*19q* co-deleted, *H3K27M*-mutant, *SHH* and *p53* status, etc
		Non-CNS	AFP, CA-125, CEA, hCG, PSA, etc. Vary by tumor site
	Extent	NOS	Extension of the involvement; vary by tumor site
	Metastasis	CNS	NA
		Non-CNS	Brain/bone/lung/liver metastasis available after 2016
	Stage	CNS	NA
		Non-CNS	AJCC T/M/N staging and staging group
	Lymph node status	CNS	NA
		Non-CNS	Number of examined and positive regional nodes
Treatment	Surgery	NOS	Specifies if receiving the first course surgical treatment; operation type; extent of resection
	Lymph node surgery	CNS	NA
		Non-CNS	Number of regional lymph nodes removed
	Radiotherapy	NOS	Specifies if receiving the first course of radiotherapy; radiotherapy type
	Chemotherapy	NOS	Specifies if receiving the first course of chemotherapy
Outcomes	Status	NOS	Status at the last time reporting to the registry
	Follow-up time	NOS	Survival time or follow-up time

*AFP* alpha-fetoprotein, *AJCC* American Joint Committee on Cancer, *CA-125* cancer antigen 125, *CEA* carcinoembryonic antigen, *CNS* central nervous system, *hCG* human chorionic gonadotrophin, *H3K27M* histone 3 lysine 27, *ICD-O-3* International Classification of Diseases for Oncology, *IDH* isocitrate dehydrogenase, *NOS* not otherwise specified, *NA* not applicable, *PSA* prostate specific antigen, *SHH* sonic hedgehog, *WHO* World Health Organization

## Statistical methods

### Logistic regression model

The logistic function was developed during the nineteenth century to describe population expansion and the progress of autocatalytic chemical processes [16]. The binary logistic regression model is one of the most extensively used prediction models in medicine to predict the occurrence of a clinical event, such as disease, recurrence, mortality, or recovery. A closed exponential formula is applied to calculate the probability of an occurrence based on a set of parameters [17]. Odds ratios (*OR*s), which correspond to the probabilities of binary outcomes, are commonly reported in the medical literature [18]. Logistic regression analysis is a type of generalized linear model [19, 20] that is frequently examined in SEER-based studies for short-term survival analysis (less than 1 year) [21, 22]. As a measure of short-term surgical outcome, the 1-month survival rate has been widely used for the evaluation of treatment effectiveness [23]. For example, it has been reported that logistic regression was used to identify two covariables associated with 1-month mortality in 5428 surgically treated brain tumor patients [24]. The authors found that pediatric patients under 1 year old had a significantly higher risk of 1-month mortality [*OR* = 5.9, 95% confidence interval (CI) 3.4–10.4]. Identifying compatible individuals for a certain medication is also an efficient technique for implementing precision treatment from a medical standpoint. Whether or not patients should undergo treatment has been one of the hot topics in cancer research [25]. In one study, external-beam radiation was independently related to higher 1-year survival in postoperative patients with gallbladder cancer. In addition, patients at a younger age with tumor spread beyond the serosa, intermediate to poorly differentiated tumors, and lymph node metastases are more likely to have received external-beam radiation treatment (*OR* > 1) [26]. Logistic regression has become a standard statistical tool for SEER-based research, such as risk assessments in the presence of synchronous metastases. In particular, the associations of age and sex with the presence of synchronous brain metastases (SBMs) have been studied intensively [20, 27]. Indeed, logistic regression is a widely utilized method for estimating propensity scores by regressing the binary treatment or exposure indicator variable on pretreatment variables [28].

### Cox proportional-hazards model

David Cox established the proportional-hazards model in 1972 to evaluate how multiple covariates influence the time to failure of a system [29]. Cox proportional-hazards regression is one of the most commonly used regression methods for survival analysis and is used to correlate multiple risk variables or exposure types with survival time [30, 31]. In most cases, different groups are compared based on their hazards, and thus the hazard ratio (*HR*) is used as it is equivalent to an *OR* in the framework of logistic regression analysis [32]. The SEER program provides long-term follow-up outcome data that are regularly updated, making it ideal for Cox regression analyses. For example, the primary tumor of the triple-negative subtype (vs. hormone receptor^+^/HER2^−^: *HR* = 1.98, 95%CI 1.56–2.50) had the highest adjusted risk of death in multivariable Cox regression for all-cause mortality among breast cancer patients with SBMs from the SEER database [33]. The best treatment modality for patients with malignancies has been intensively studied by Cox regression analyses. Pausch et al. [34] found that chemotherapy and cancer-directed surgery are significant protective prognostic factors (*HR* < 1, *P* < 0.05) for patients with oligometastatic pancreatic ductal adenocarcinoma (PDAC). In addition, several SEER-based studies reported the application of Cox proportional-hazards models to evaluate the associations of examined lymph node count [35], socioeconomic status [36], insurance status [37], marital status [38], and other clinicopathological variables with the prognosis of cancer patients. Within this class of analysis, the Kaplan–Meier method has been used to estimate survival, and a stratified log-rank test was used to assess differences in survival [39]. It should be noted that a restricted cubic spline (RCS) function is required when nonlinearities appear [20, 40].

### Competing-risks model

Prognostic models should consider competing events because they affect assessments of the impact of the event of interest and, thus, the benefit of an intervention [41]. The competing-risks data inherent in medical research can be analyzed using proportional cause-specific hazard and proportional subdistribution hazard (SDH) models [42]. The Fine-Gray regression method introduced by Fine and Gray [43] in 1999 is one of the most widely used models for proportional-hazards modeling of the SDH. SDH models are considered to be more desirable for direct evaluations of actual hazards, and, therefore, they can be used for prognosis assessment and in medical decision-making [44]. Although the cause of mortality could be difficult to define accurately, the SEER program divides the cause of death into cancer-specific death and other causes of death. These two groups can be set as the main or competing events. Accordingly, Li et al. [45] used the Cox regression model to perform a SEER-based analysis and revealed that the risk of other causes of death increased with age, which was supported by the findings from a competing-risks model, which indicated an association between an increased risk of all-cause death and advanced age. Another SEER-based study found that the prognosis was worse in Medicaid patients than in insured patients (subhazard ratio = 1.87, 95%CI 1.72–2.04, *P* < 0.0001) based on a Fine-Gray competing-risks model. It should be noted that the cumulative incidence function is typically used instead of Kaplan–Meier curves in the case of competing risks since the Kaplan–Meier estimator often overestimates the cumulative incidence in the presence of competing risks [46, 47].

### RCS model

As reported previously [48], cubic spline functions are computationally easy to use, and they can define various geometries if sufficient knots are included. RCSs are the cubic splines that are constrained to be linear in the tail of a distribution developed by Stone and Koo [49]. Herndon and Harrell [50] demonstrated that in a homogeneous setting (i.e., with no covariables), the RCS hazard function has enough flexibility to describe a wide range of hazard-function shapes without becoming computationally intractable. However, only a few continuous variables appropriate for RCS analysis could be obtained from the SEER program [20].

### Poisson regression model

Poisson regression is one of the generalized linear models that is used when the dependent variable is described by the count data [51]. It is suitable for summarizing relative risks and analyzing complicated interactions among factors. In addition, Poisson regression can be broadly applied to the estimation of disease incidence based on assumptive etiological processes of exposure or disease-related features in a population [52]. For example, Tsikitis et al. [53] used Poisson regression to evaluate trends in incidence rates of gastrointestinal neuroendocrine tumors over time, with the year of diagnosis as a continuous variable. In addition, Muskens et al. [54] utilized a Poisson regression model to compare age-adjusted incidence rates of pediatric glioma and medulloblastoma in a multiple-variable analysis. A Poisson regression model was also used in a SEER-based study to examine the characteristics of Wilms tumors that impacted lymph node density [55].

### Nomogram

A nomogram provides an easy-to-interpret graphical depiction of a statistical prediction model that can predict the probability of a particular clinical event [56]. Because of their ability to provide personalized predictions, nomograms can be used to identify high-risk populations and stratify patients in clinical trials. The combination of a user-friendly interface with easy online access has led to their widespread acceptance by both oncologists and patients [57]. Iasonos et al. [56] described the following steps for constructing a nomogram for cancer patients: (1) screening patients; (2) determination of outcome; (3) screening significant predictors; (4) construction of a nomogram; (5) validation of the nomogram; and (6) interpretation of the nomogram. The nomograms in previous SEER-based studies have primarily been constructed based on logistic regression, Cox regression, and competing-risks models. Pan et al. [58] applied a Cox regression model to screen 9 prognostic factors for the overall survival (OS) of patients with inflammatory breast cancer. They developed a nomogram that was internally and externally validated to predict the 1-, 3-, and 5-year OS rates for patients with inflammatory breast cancer. Wu et al. [59] used a logistic regression model to identify 3 independent factors for the construction of a nomogram that can predict the lymph node metastatic status of breast mucinous carcinoma. The nomogram can also be constructed using a competing-risks model to predict the survival of patients with node-negative localized renal cell carcinoma [60]. In addition, nomograms can be used for the clinical risk stratification of malignancies [61, 62].

### Regression using the least absolute shrinkage and selection operator (LASSO)

The LASSO was developed by Tibshirani [63]. The merit of this method is that it can reduce certain coefficients and sets other than zero in order to keep the best characteristics of both subset selection and ridge regression. The LASSO regression was later proved mathematically by Zhao et al. [64]. It can be used in SEER-based studies to identify predictors for a binary outcome. Che et al. [20, 27] used LASSO regression models to identify predictors associated with the presence of SBMs in patients with breast cancer. The prognostic variables impacting OS and cancer-specific survival in patients with pancreatic adenosquamous carcinoma were also identified using LASSO analyses [65].

### Artificial intelligence (AI)

There are two types of AI applications in medicine: virtual and physical. Machine learning is a virtual type of AI [66], which is implemented by mathematical algorithms that increase learning ability via experience [67]. There is an increasing and irreversible trend of discipline convergence between medical science and AI [68]. Yu et al. [69] developed the DeepSurv model, which combines machine learning with a multilayer neural network to predict the survival of patients with rectal adenocarcinoma. They showed that the AI-based prediction model had a higher C-index and better predictive capacity than traditional Cox regression survival analysis [69]. Senders et al. [70] further constructed an AI-based online calculator for predicting the survival rates of patients with glioblastoma. A comparison of the prediction accuracies of 15 statistical and machine-learning methods revealed that the accelerated failure-time model performed the best [70]. However, whether AI provides superior performance in the field of medicine requires more investigation.

### Joinpoint regression model

Kim et al. [71] developed a joinpoint regression model for analyzing the changes in cancer mortality and incidence trends. They further used the grid-search method to fit the regression function. Their algorithm determined the calendar year (as the name “joinpoints” implies) during which there were significant annual percentage changes by choosing the best-fitting log-linear regression model that needed the fewest number of joinpoints to fit the data. In addition, Lim et al. [72] used a joinpoint regression model to analyze incidence and mortality data of patients with thyroid cancer in the US obtained during 1974–2013 from 9 registries in the SEER database to analyze the true incidence and mortality rates. They found that the overall incidence and mortality rates of thyroid cancer increased annually by 3% and 1.1%, respectively. Some studies further suggest that the joinpoint regression model is a topical ecological research method in SEER-based studies [73, 74].

### Propensity-score matching (PSM)

Rosenbaum and Rubin [75] developed the PSM method for constructing a small control group with a covariate distribution comparable to the distribution of the treatment group in an observational study. Propensity-score analyses have been shown to be able to successfully imitate various randomized clinical trials that assess diverse target groups. They also showed that this method could eliminate bias in comparisons between treated and control populations [76]. PSM has become a well-established method for estimating causal treatment effects [77]. The most popular PSM technique uses 1:1 nearest-neighbor matching (also known as greedy matching), in which each person who received treatment A is evaluated sequentially to another person who received treatment B with the closest propensity-score matched, typically within a predetermined bound on the closest propensity scores [78]. The influence of treatment (surgery, chemotherapy, or radiotherapy) on the prognosis of patients with malignancies has been examined in numerous SEER-based studies using PSM [34, 79, 80]. PSM is ideal for adjusting pertinent confounding variables when studies focus on different subtypes of a particular malignancy [81].

### Other models

SEER-based studies may also employ several other research methodologies. For example, mediation analysis is typically used to identify the indirect impact of a covariate on cancer survival through one or a few mediating factors [82, 83]. Possible interactions of treatment and other variables with mortality have been explored in subgroup analysis, which could enhance the reliability of the results [84, 85]. Exploratory factor analysis based on varimax rotation was used to diminish the data set, leading to the discovery of the intricately connected structure of county-level socioeconomic status indicators [36, 86].

## Study designs

### Real-world study

The method of using a real-world study that was first introduced by Kaplan et al. [87] in 1993 involved acquiring real-world data from various sources, including electronic health records, administrative data, health insurance claims and billing data, product and disease registries, personal devices, and health applications [88, 89]. Furthermore, real-world evidence has shown that variables such as clinical settings, provider features, and health-system characteristics could affect treatment effects and outcomes [88]. As one of the most important cancer registries in the US, the SEER program collected complete and accurate data on all cancers diagnosed among the inhabitants in specified geographic regions. It is maintained with a continuous quality control and improvement program to ensure that high-quality data are obtained. Obviously, the SEER program is an important source of real-world data. Under the premise of using appropriate analytic tools and methods, SEER-based real-world studies can generate valuable real-world evidence [90]. For example, in a SEER-based investigation, Yuan et al. [91] used a real-world study design and discovered that the overall mortality risk was higher for focal treatment than for active surveillance or watchful waiting, indicating that the latter could offer OS benefits. Nevertheless, a careful examination of the literature revealed that there have been very few real-world studies that make use of the SEER database. We hope that this review will raise awareness of the availability of real-world data from the SEER program.

### Ecological study

Being one of the most fundamental types of observational studies, ecological study is ideally suited for SEER-based research. This study examines groups of individuals who were typically categorized according to their geographic location or chronological associations [92, 93]. It can also estimate the prevalence of diseases in a community by assigning a single exposure level for each unique group. An elegant example of a SEER-based ecological study involved a description of incidence trends and disparities in cancers related to *Helicobacter pylori* reported by Lai et al. [94]. They found that the incidence of *Helicobacter pylori*-related cancers showed a significant downward trend from 2000 to 2019 and identified the racial/ethnic and geographic disparities in incidence rates. In addition, the demographic disparities in the incidence rates of SBMs [95], gliomas [74], and thyroid cancer [72] have been reported using this approach. The Rate Session in SEER*Sat software can be used to obtain the data when demographic covariables are considered in the exposure indicator and the outcome is a cancer diagnosis.

### Proportional mortality ratio (PMR) study

The SEER database contains information obtained from state-issued death certificates about the causes of death [96], and data collected from the US Census Bureau can be used to compute mortality statistics. These data are used for the PMR studies. The PMR and standardized mortality ratio (SMR) are the epidemiological outcomes of this type of study. The two ratios represent the proportions of cause-specific deaths relative to all deaths for each exposure group [97]. Long-term follow-up analysis revealed that PMR is likely to be higher for cardiovascular disease than for classic Hodgkin lymphoma among patients with stage I and stage II classic Hodgkin lymphoma [98]. By analyzing the relative risk of mortality compared with all people using the SMR, Zaorsky et al. [99] identified variations in the risks of death from index and non-index cancers among primary cancer locations. It should be cautioned that assessments by PMR may not always be reliable due to a lack of information about the populations at risk. Therefore, even though the denominator or numerator of the ratio is skewed, it is suggested that SMRs should be used instead of PMRs. In fact, the frequency of using SMR is higher than that of PMR in SEER-based studies. The corresponding statistical data can be obtained using SEER*Sat software under the MP-SIR session.

### Cohort study

The term “cohort” was first used in medical applications in 1935 by Wade Hampton Frost, an epidemiologist who studied age-specific mortality rates [100]. According to the field of epidemiology, nowadays the term refers to a group of people with defined characteristics who are followed up for the assessment of incidence or mortality from a specific cause of death, all causes of death, or some other outcomes [101]. In a typical cohort study, a group of participants is followed over time. As an exemplary cohort study, Pausch et al. [34] conducted a SEER-based study and discovered a relationship between cancer-directed surgery and a better prognosis of patients with PDAC. Based on the final follow-up date for recorded survival on December 31, 2015, the authors found that cancer-directed surgery significantly increased the median OS of patients with PDAC from 5 to 10 months. It should be noted that the SEER-based study has a tendency to be imbalanced in baseline characteristics, and propensity score matching (PSM) can be utilized to reduce the associated bias in this situation. The SEER data used in cohort, case–control, and case-series studies can be obtained using SEER*Sat software under the Case-Listing session.

### Cross-sectional study

Cross-sectional study is conducted at a specific point in time or spanning a relatively short time frame. These studies are often used to estimate the prevalence of an outcome of interest in a specific population, especially for planned public health strategies. Together with outcome information, data on individual characteristics such as exposure to risk factors can be obtained from these studies [101]. Depending on whether the results are evaluated for potential association with risk variables or exposures, cross-sectional studies can be descriptive or analytical [102]. In a previous cross-sectional study, we recorded the prevalence of SBMs and analyzed the relationship between SBMs and clinicopathological data in midlife patients [27]. The outcome was the presence or absence of SBMs, whereas the exposure factors were patient age, sex, race, marital status, and other covariables. Specifically, we analyzed the clinicopathological data of patients, assessed their SBM status, and evaluated the outcomes and exposure data simultaneously. Given that the cross-sectional studies estimate prevalence rates, they are particularly useful for analyzing the burden of a disease or condition for planning health care services. The data can be obtained using SEER*Sat software under Survival and Case-Listing session.

### Case–control study

Case–control study has been widely used to address significant public health issues [103]. This design was first applied in the breast cancer study by Lane-Claypon in 1926 [104], leading to the conclusion that a low fertility rate increases the risk of breast cancer. Because of the inherent characteristics of the SEER-Medicare database and the SEER database, the former is more suitable for case–control studies. However, researchers will need to further evaluate whether SEER-based studies use this study design appropriately.

### Case-series study

A case series includes multiple individuals across time who were diagnosed with the same disease or received the same treatment [105]. Case-series studies are subsets of descriptive studies that do not explore the effectiveness of hypothesized treatments [106]. This characteristic makes case series a relatively efficient and cost-effective approach because it does not use randomization or comparison groups. However, despite being one of the most representative large databases of tumors in North America, SEER-based case-series investigations are uncommon. The description of the defining characteristics of patients with malignant thyroid teratomas would be a typical SEER-based case series. A study using 8 patients with malignant thyroid teratoma indicated a high rate of extrathyroidal extension and nodal involvement, as well as easy recurrence and metastases, which are characteristic features of these neoplasms [107]. The main goal of a case-series study is to generate hypotheses that can be further validated by rigorous statistical methods.

## Conclusions and perspectives

The effective use of the SEER database for cancer research depends on the appropriate application of study designs and statistical models. The purpose of this review is to assist clinical researchers in understanding the types of advanced statistical modeling methods and study designs. Appropriate use of the SEER database can ensure that correct research conclusions are drawn and maximize the benefits to clinicians and patients. Through recently published exemplary cases, we have shown that there are diverse statistical methodologies and study designs that can be applied to SEER-based research. It is important to point out that a SEER-based study usually has a complex integrated design and involves various statistical methods. It is hoped that the structural framework of this review will help readers obtain relevant data and better understand and choose their study designs and methods.

The types of study designs used in the SEER-based studies have been progressively refined [108]. The SEER program currently records information on around 400,000 cancer cases annually. The volume of SEER data has been growing fast [109]. The analysis of greater volumes of big data with higher dimensionality necessitates novel ideas and methodologies. The present review offers several implications for data collection, standardization of analysis, and cancer surveillance for national and military health systems surveillance institutes.

## Data Availability

The data used in the study can be found at: https://seer.cancer.gov.

## Abbreviations

AI: Artificial intelligence
CI: Confidence interval
LASSO: Least absolute shrinkage and selection operator
*OR*: Odds ratio
OS: Overall survival
PDAC: Pancreatic ductal adenocarcinoma
PMR: Proportional mortality ratio
PSM: Propensity score matching
RCS: Restricted cubic spline
SEER: Surveillance, Epidemiology, and End Results
SDH: Subdistribution hazard
SBMs: Synchronous brain metastases
SMR: Standardized mortality ratio
US: United States

## References

[1] Park HS, Lloyd S, Decker RH, Wilson LD, Yu JB (2012). Overview of the Surveillance, Epidemiology, and End Results database: evolution, data variables, and quality assurance. Curr Probl Cancer.

[2] Malmgren JA, Calip GS, Atwood MK, Mayer M, Kaplan HG (2020). Metastatic breast cancer survival improvement restricted by regional disparity: Surveillance, Epidemiology, and End Results and institutional analysis: 1990 to 2011. Cancer.

[3] Sasaki K, Jabbour E, Short NJ, Jain N, Ravandi F, Pui CH (2021). Acute lymphoblastic leukemia: a population-based study of outcome in the United States based on the Surveillance, Epidemiology, and End Results (SEER) database, 1980–2017. Am J Hematol.

[4] Miller KD, Nogueira L, Devasia T, Mariotto AB, Yabroff KR, Jemal A (2022). Cancer treatment and survivorship statistics, 2022. CA Cancer J Clin.

[5] Mehta RS, Lenzner D, Argiris A (2012). Race and health disparities in patient refusal of surgery for early-stage non-small cell lung cancer: a SEER cohort study. Ann Surg Oncol.

[6] Zavala VA, Bracci PM, Carethers JM, Carvajal-Carmona L, Coggins NB, Cruz-Correa MR (2021). Cancer health disparities in racial/ethnic minorities in the United States. Br J Cancer.

[7] Daly MC, Paquette IM (2019). Surveillance, Epidemiology, and End Results (SEER) and SEER-medicare databases: use in clinical research for improving colorectal cancer outcomes. Clin Colon Rectal Surg.

[8] Brar G, Greten TF, Graubard BI, Mcneel TS, Petrick JL, Mcglynn KA (2020). Hepatocellular carcinoma survival by etiology: a SEER-Medicare database analysis. Hepatol Commun.

[9] Barzi A, Zhou K, Wang S, Dodge JL, El-Khoueiry A, Setiawan VW (2021). Etiology and outcomes of hepatocellular carcinoma in an ethnically diverse population: the multiethnic cohort. Cancers (Basel).

[10] Tonidandel S, King EB, Cortina JM (2018). Big data methods: leveraging modern data analytic techniques to build organizational science. Organ Res Methods.

[11] Hasan MM, Popp J, Oláh J (2020). Current landscape and influence of big data on finance. J Big Data.

[12] Zhang L, Wang H, Li Q, Zhao MH, Zhan QM (2018). Big data and medical research in China. BMJ.

[13] Alharthi A, Krotov V, Bowman M (2017). Addressing barriers to big data. Bus Horizons.

[14] Yang J, Li Y, Liu Q, Li L, Feng A, Wang T (2020). Brief introduction of medical database and data mining technology in big data era. J Evid Based Med.

[15] Wu WT, Li YJ, Feng AZ, Li L, Huang T, Xu AD (2021). Data mining in clinical big data: the frequently used databases, steps, and methodological models. Mil Med Res.

[16] Boateng EY, Abaye DA (2019). A review of the logistic regression model with emphasis on medical research. JDAIP.

[17] Sur P, Candès EJ (2019). A modern maximum-likelihood theory for high-dimensional logistic regression. Proc Natl Acad Sci U S A.

[18] Norton EC, Dowd BE, Maciejewski ML (2018). Odds ratios-current best practice and use. JAMA.

[19] Shipe ME, Deppen SA, Farjah F, Grogan EL (2019). Developing prediction models for clinical use using logistic regression: an overview. J Thorac Dis.

[20] Che W, Wang Y, Wang X, Lyu J (2022). Association between age and the presence and mortality of breast cancer synchronous brain metastases in the United States: a neglected SEER analysis. Front Public Health.

[21] Lorimer PD, Motz BM, Watson M, Trufan SJ, Prabhu RS, Hill JS (2019). Enteral feeding access has an impact on outcomes for patients with esophageal cancer undergoing esophagectomy: an analysis of SEER-Medicare. Ann Surg Oncol.

[22] Bartek J, Dhawan S, Thurin E, Alattar A, Gulati S, Rydenhag B (2020). Short-term outcome following surgery for rare brain tumor entities in adults: a Swedish nation-wide registry-based study and comparison with SEER database. J Neurooncol.

[23] Chang W, Wei Y, Ren L, Jian M, Chen Y, Chen J (2020). Short-term and long-term outcomes of robotic rectal surgery-from the real word data of 1145 consecutive cases in China. Surg Endosc.

[24] Hankinson TC, Dudley RWR, Torok MR, Patibandla MR, Dorris K, Poonia S (2016). Short-term mortality following surgical procedures for the diagnosis of pediatric brain tumors: outcome analysis in 5533 children from SEER, 2004–2011. J Neurosurg Pediatr.

[25] Wu C, Zhao Y, Zhang Y, Yang Y, Su W, Yang Y (2022). Gut microbiota specifically mediates the anti-hypercholesterolemic effect of berberine (BBR) and facilitates to predict BBR’s cholesterol-decreasing efficacy in patients. J Adv Res.

[26] Hyder O, Dodson RM, Sachs T, Weiss M, Mayo SC, Choti MA (2014). Impact of adjuvant external beam radiotherapy on survival in surgically resected gallbladder adenocarcinoma: a propensity score-matched Surveillance, Epidemiology, and End Results analysis. Surgery.

[27] Che W, Wang Y, Wang X, Lyu J (2022). Midlife brain metastases in the United States: Is male at risk?. Cancer Med.

[28] Coffman DL, Zhou J, Cai X (2020). Comparison of methods for handling covariate missingness in propensity score estimation with a binary exposure. BMC Med Res Methodol.

[29] Cox DR (1972). Regression models and life-tables. J R Stat Soc B.

[30] Moolgavkar SH, Chang ET, Watson HN, Lau EC (2018). An assessment of the Cox proportional hazards regression model for epidemiologic studies. Risk Anal.

[31] He QL, Gao SW, Qin Y, Huang RC, Chen CY, Zhou F (2022). Gastrointestinal dysfunction is associated with mortality in severe burn patients: a 10-year retrospective observational study from South China. Mil Med Res.

[32] Kalbfleisch JD, Schaubel DE (2023). Fifty years of the cox model. Annu Rev Stat Appl.

[33] Martin AM, Cagney DN, Catalano PJ, Warren LE, Bellon JR, Punglia RS (2017). Brain metastases in newly diagnosed breast cancer: a population-based study. JAMA Oncol.

[34] Pausch TM, Liu X, Cui J, Wei J, Miao Y, Heger U (2022). Survival benefit of resection surgery for pancreatic ductal adenocarcinoma with liver metastases: a propensity score-matched SEER database analysis. Cancers (Basel).

[35] Bhanvadia RR, Rodriguez J, Bagrodia A, Eggener SE (2019). Lymph node count impacts survival following post-chemotherapy retroperitoneal lymphadenectomy for non-seminomatous testicular cancer: a population-based analysis. BJU Int.

[36] Che W, Ma W, Lyu J, Wang X (2021). Socioeconomic status and adult gliomas mortality risk: an observational study based on SEER data. World Neurosurg.

[37] Saraswathula A, Megwalu UC (2018). Insurance status and survival of patients with salivary gland cancer. Otolaryngol Head Neck Surg.

[38] Zhang SL, Wang WR, Liu ZJ, Wang ZM (2019). Marital status and survival in patients with soft tissue sarcoma: a population-based, propensity-matched study. Cancer Med.

[39] Zabor EC, Radivoyevitch T, Singh AD, Kilic E, de Klein JEMM, Kalirai H (2021). Conditional survival in uveal melanoma. Ophthalmol Retina.

[40] Kitajima K, Igeta M, Kuyama J, Kawahara T, Suga T, Otani T (2023). Novel nomogram developed for determining suitability of metastatic castration-resistant prostate cancer patients to receive maximum benefit from radium-223 dichloride treatment-Japanese Ra-223 Therapy in Prostate Cancer using Bone Scan Index (J-RAP-BSI) Trial. Eur J Nucl Med Mol Imaging.

[41] Wolbers M, Koller MT, Witteman JCM, Steyerberg EW (2009). Prognostic models with competing risks: methods and application to coronary risk prediction. Epidemiology.

[42] Latouche A, Allignol A, Beyersmann J, Labopin M, Fine JP (2013). A competing risks analysis should report results on all cause-specific hazards and cumulative incidence functions. J Clin Epidemiol.

[43] Fine JP, Gray RJ (1999). A proportional hazards model for the subdistribution of a competing risk. J Am Stat Assoc.

[44] Li Y, Sun L, Burstein DS, Getz KD (2022). Considerations of competing risks analysis in cardio-oncology studies: JACC: cardiooncology state-of-the-art review. JACC CardioOncol.

[45] Li X, Liu Z, Ye Z, Gou S, Wang C (2018). Impact of age on survival of patients with pancreatic cancer after surgery: analysis of SEER data. Pancreatology.

[46] Gooley TA, Leisenring W, Crowley J, Storer BE (1999). Estimation of failure probabilities in the presence of competing risks: new representations of old estimators. Stat Med.

[47] Yang J, Pan Z, He Y, Zhao F, Feng X, Liu Q (2019). Competing-risks model for predicting the prognosis of penile cancer based on the SEER database. Cancer Med.

[48] Wold S (1974). Spline functions in data analysis. Technometrics.

[49] Stone CJ, Koo CY. Additive splines in statistics. In: Proceedings of the American Statistical Association. Washington DC; 1985. p. 45–8.

[50] Herndon JE, Harrell FE (1990). The restricted cubic spline hazard model. Commun Stat-Theor M.

[51] Frome EL, Kutner MH, Beauchamp JJ (1973). Regression analysis of poisson-distributed data. J Am Stat Assoc.

[52] Frome EL, Checkoway H (1985). Use of poisson regression models in estimating incidence rates and ratios. Am J Epidemiol.

[53] Tsikitis VL, Wertheim BC, Guerrero MA (2012). Trends of incidence and survival of gastrointestinal neuroendocrine tumors in the United States: a seer analysis. J Cancer.

[54] Muskens IS, Feng Q, Francis SS, Walsh KM, Mckean-Cowdin R, Gauderman WJ (2020). Pediatric glioma and medulloblastoma risk and population demographics: a Poisson regression analysis. Neurooncol Adv..

[55] Walker JP, Johnson JS, Eguchi MM, Saltzman AF, Cockburn M, Cost NG (2020). Factors affecting lymph node sampling patterns and the impact on survival of lymph node density in patients with Wilms tumor: a Surveillance, Epidemiology, and End Result (SEER) database review. J Pediatr Urol.

[56] Iasonos A, Schrag D, Raj GV, Panageas KS (2008). How to build and interpret a nomogram for cancer prognosis. J Clin Oncol.

[57] Bandini M, Marchioni M, Pompe RS, Tian Z, Gandaglia G, Fossati N (2018). First North American validation and head-to-head comparison of four preoperative nomograms for prediction of lymph node invasion before radical prostatectomy. BJU Int.

[58] Pan X, Yang W, Chen Y, Tong L, Li C, Li H (2019). Nomogram for predicting the overall survival of patients with inflammatory breast cancer: a SEER-based study. Breast.

[59] Wu SL, Gai JD, Yu XM, Mao X, Jin F (2022). A novel nomogram and risk classification system for predicting lymph node metastasis of breast mucinous carcinoma: a SEER-based study. Cancer Med.

[60] Kutikov A, Egleston BL, Wong YN, Uzzo RG (2010). Evaluating overall survival and competing risks of death in patients with localized renal cell carcinoma using a comprehensive nomogram. J Clin Oncol.

[61] Yan B, Su BB, Bai DS, Qian JJ, Zhang C, Jin SJ (2021). A practical nomogram and risk stratification system predicting the cancer-specific survival for patients with early hepatocellular carcinoma. Cancer Med.

[62] Wang Y, Zheng Q, Jia B, An T, Zhao J, Wu M (2020). Effects of surgery on survival of early-stage patients with SCLC: propensity score analysis and nomogram construction in SEER database. Front Oncol.

[63] Tibshirani R (1996). Regression shrinkage and selection via the Lasso. J R Stat Soc B.

[64] Zhao P, Yu B (2006). On model selection consistency of Lasso. J Mach Learn Res.

[65] Yang Z, Shi G, Zhang P (2022). Development and validation of nomograms to predict overall survival and cancer-specific survival in patients with pancreatic adenosquamous carcinoma. Front Oncol.

[66] Peng HT, Siddiqui MM, Rhind SG, Zhang J, da Luz LT, Beckett A (2023). Artificial intelligence and machine learning for hemorrhagic trauma care. Mil Med Res.

[67] Haug CJ, Drazen JM (2023). Artificial intelligence and machine learning in clinical medicine, 2023. N Engl J Med.

[68] Topol EJ (2019). High-performance medicine: the convergence of human and artificial intelligence. Nat Med.

[69] Yu H, Huang T, Feng B, Lyu J (2022). Deep-learning model for predicting the survival of rectal adenocarcinoma patients based on a Surveillance, Epidemiology, and End Results analysis. BMC Cancer.

[70] Senders JT, Staples P, Mehrtash A, Cote DJ, Taphoorn MJB, Reardon DA (2020). An online calculator for the prediction of survival in glioblastoma patients using classical statistics and machine learning. Neurosurgery.

[71] Kim HJ, Fay MP, Feuer EJ, Midthune DN (2000). Permutation tests for joinpoint regression with applications to cancer rates. Stat Med.

[72] Lim H, Devesa SS, Sosa JA, Check D, Kitahara CM (2017). Trends in thyroid cancer incidence and mortality in the United States, 1974–2013. JAMA.

[73] Guo F, Kuo YF, Shih YCT, Giordano SH, Berenson AB (2018). Trends in breast cancer mortality by stage at diagnosis among young women in the United States. Cancer.

[74] Lin D, Wang M, Chen Y, Gong J, Chen L, Shi X (2021). Trends in Intracranial Glioma Incidence and Mortality in the United States, 1975–2018. Front Oncol.

[75] Rosenbaum PR, Rubin DB (1983). The central role of the propensity score in observational studies for causal effects. Biometrika.

[76] Staffa SJ, Zurakowski D (2018). Five steps to successfully implement and evaluate propensity score matching in clinical research studies. Anesth Analg.

[77] Simoneau G, Pellegrini F, Debray TP, Rouette J, Muñoz J, Platt RW (2022). Recommendations for the use of propensity score methods in multiple sclerosis research. Mult Scler.

[78] Thomas L, Li F, Pencina M (2020). Using propensity score methods to create target populations in observational clinical research. JAMA.

[79] Qi L, Wan L, Ren X, Zhang W, Tu C, Li Z (2020). The role of chemotherapy in extraskeletal osteosarcoma: a propensity score analysis of the surveillance epidemiology and end results (SEER) database. Med Sci Monit.

[80] Lim YJ, Song C, Kim JS (2017). Improved survival with postoperative radiotherapy in thymic carcinoma: a propensity-matched analysis of Surveillance, Epidemiology, and End Results (SEER) database. Lung Cancer.

[81] Liu Z, Zeng W, Huang L, Wang Z, Wang M, Zhou L (2018). Prognosis of FTC compared to PTC and FVPTC: findings based on SEER database using propensity score matching analysis. Am J Cancer Res.

[82] Lin SW, Anisa KN (2021). Effects of socioeconomic status on cancer patient survival: counterfactual event-based mediation analysis. Cancer Causes Control.

[83] Leapman MS, Dinan M, Pasha S, Long J, Washington SL, Ma X (2022). Mediators of racial disparity in the use of prostate magnetic resonance imaging among patients with prostate cancer. JAMA Oncol.

[84] Jiang X, Yan M (2021). Surgical treatment for improved 1-year survival in patients with primary cardiac sarcoma. Anatol J Cardiol.

[85] Liu X, Wang C, Feng Y, Shen C, He T, Wang Z (2022). The prognostic role of surgery and a nomogram to predict the survival of stage IV breast cancer patients. Gland Surg.

[86] Knoble NB, Alderfer MA, Hossain MJ (2016). Socioeconomic status (SES) and childhood acute myeloid leukemia (AML) mortality risk: analysis of SEER data. Cancer Epidemiol.

[87] Kaplan NM, Sproul LE, Mulcahy WS (1993). Large prospective study of ramipril in patients with hypertension. CARE Investig Clin Ther.

[88] Sherman RE, Anderson SA, Dal Pan GJ, Gray GW, Gross T, Hunter NL (2016). Real-world evidence-what is it and what can it tell us. N Engl J Med.

[89] Corrigan-Curay J, Sacks L, Woodcock J (2018). Real-world evidence and real-world data for evaluating drug safety and effectiveness. JAMA.

[90] Fang Y, He W, Wang H, Wu M (2020). Key considerations in the design of real-world studies. Contemp Clin Trials.

[91] Yuan QM, Lin TH, Jin K, Qiu S, Zhou XH, Jin D (2022). The comparison of survival between active surveillance or watchful waiting and focal therapy for low-risk prostate cancer: a real-world study from the SEER database. Asian J Androl.

[92] Morgenstern H (1995). Ecologic studies in epidemiology: concepts, principles, and methods. Annu Rev Public Health.

[93] Mackenbach JP (1995). Public health epidemiology. J Epidemiol Community Health.

[94] Lai Y, Shi H, Wang Z, Feng Y, Bao Y, Li Y (2022). Incidence trends and disparities in Helicobacter pylori related malignancy among US adults, 2000–2019. Front Public Health.

[95] Che W, Liu J, Fu T, Wang X, Lyu J (2022). Recent trends in synchronous brain metastasis incidence and mortality in the United States: ten-year multicenter experience. Curr Oncol.

[96] Horn SR, Stoltzfus KC, Mackley HB, Lehrer EJ, Zhou S, Dandekar SC (2020). Long-term causes of death among pediatric patients with cancer. Cancer.

[97] Monson RR (1974). Analysis of relative survival and proportional mortality. Comput Biomed Res.

[98] Lu Z, Teng Y, Ning X, Wang H, Feng W, Ou C (2022). Long-term risk of cardiovascular disease mortality among classic Hodgkin lymphoma survivors. Cancer.

[99] Zaorsky NG, Churilla TM, Egleston BL, Fisher SG, Ridge JA, Horwitz EM (2017). Causes of death among cancer patients. Ann Oncol.

[100] Comstock GW (2001). Cohort analysis: W.H. Frost's contributions to the epidemiology of tuberculosis and chronic disease. Soz Praventivmed.

[101] Levin KA (2006). Study design III: cross-sectional studies. Evid Based Dent.

[102] Wang X, Cheng Z (2020). Cross-sectional studies: strengths, weaknesses, and recommendations. Chest.

[103] Dey T, Mukherjee A, Chakraborty S (2020). A practical overview of case-control studies in clinical practice. Chest.

[104] Macki M, Air EL (2019). Commentary: what is a case control study?. Neurosurgery.

[105] Kooistra B, Dijkman B, Einhorn TA, Bhandari M (2009). How to design a good case series. J Bone Joint Surg Am.

[106] Dekkers OM, Egger M, Altman DG, Vandenbroucke JP (2012). Distinguishing case series from cohort studies. Ann Intern Med.

[107] Vuong HG, Nguyen TPX, Ngo HTT, Hassell L, Kakudo K (2021). Malignant thyroid teratoma: an integrated analysis of case series/case reports. Endocr Relat Cancer.

[108] Thiese MS (2014). Observational and interventional study design types; an overview. Biochem Med (Zagreb).

[109] Duggan MA, Anderson WF, Altekruse S, Penberthy L, Sherman ME (2016). The surveillance, epidemiology and end results (SEER) program and pathology: towards strengthening the critical relationship. Am J Surg Pathol.

